# Development of Laser-Based Powder Bed Fusion Process Parameters and Scanning Strategy for New Metal Alloy Grades: A Holistic Method Formulation

**DOI:** 10.3390/ma11122356

**Published:** 2018-11-22

**Authors:** Elena Bassoli, Antonella Sola, Mattia Celesti, Sandro Calcagnile, Carlo Cavallini

**Affiliations:** 1 Department of Engineering “Enzo Ferrari”, University of Modena and Reggio Emilia, Via P. Vivarelli, 10, 41125 Modena, Italy; antonella.sola@unimore.it; 2 Metal Additive Research Centre, HPE s.r.l., Via R. Dalla Costa, 620, 41122 Modena, Italy; mcelesti@hpe.eu (M.C.); CCavallini@hpe.eu (C.C.); 3 3 COXA S.p.A., Via R. Dalla Costa, 620, 41122 Modena, Italy; Sandro.Calcagnile@coxa.it

**Keywords:** Laser-based powder bed fusion, processing, optimization, standardization

## Abstract

In spite of the fast growth of laser-based powder bed fusion (L-PBF) processes as a part of everyday industrial practice, achieving consistent production is hampered by the scarce repeatability of performance that is often encountered across different additive manufacturing (AM) machines. In addition, the development of novel feedstock materials, which is fundamental to the future growth of AM, is limited by the absence of established methodologies for their successful exploitation. This paper proposes a structured procedure with a complete test plan, which defines step-by-step the standardized actions that should be taken to optimize the processing parameters and scanning strategy in L-PBF of new alloy grades. The method is holistic, since it considers all the laser/material interactions in different local geometries of the build, and suggests, for each possible interaction, a specific geometry for test specimens, standard energy parameters to be analyzed through a design of experiment, and measurable key performance indicators. The proposed procedure therefore represents a sound and robust aid to the development of novel alloy grades for L-PBF and to the definition of the most appropriate processing conditions for them, independent of the specific AM machine applied.

## 1. Introduction

Powder bed fusion (PBF) is rapidly prevailing as the most important additive manufacturing (AM) technique to fabricate metal parts. Unlike conventional “subtractive” techniques such as cutting, AM produces three-dimensional parts based on computer-aided design (CAD) models by adding materials layer by layer [1].

In particular, laser-based powder bed fusion (L-PBF) uses a laser beam to induce local sintering or melting–resolidification phenomena in a powder bed that is processed layer-wise until the wanted three-dimensional geometry is achieved. Since AM technologies are “bottom-to-top” methods [2] that exploit material accumulation instead of material removal to provide the object with the required form, key advantages may be achieved in terms of design freedom, reduced time to market, and materials saving [3,4].

Nonetheless, the broader diffusion of AM is being hindered by the variability that is often observed in part quality and that becomes critical in high-value and safety-related applications, such as aerospace and biomedical devices [5,6,7]. Once the feedstock material has been assigned, possible quality issues are attributable to process parameters that are commonly adjusted by means of a trial-and-error approach. The empirical achievement of appropriate processing conditions is therefore time-consuming and expensive [8]. Moreover, this methodology is strictly material-related, in that the process parameters attributed to a specific feedstock, as well as the tentative tests performed to define such parameters, cannot be extended to other materials automatically, which means that this heuristic learning strategy must be repeated from scratch for every single powder of interest. The experimental burden associated with the adjustment of process parameters represents a serious hindrance to the development and diffusion on the market of new feedstocks for AM, and is instead a requirement for the further growth of AM [9]. In principle, the development of refined physics-based computational models is expected to reduce the number of required experimental iterations. Nonetheless, such simulations are case-specific and deeply affected by the accuracy of the input parameters. Moreover, accurate and complicated measurements are needed for their full validation [10].

At present, most of the available literature on L-PBF is dedicated to the experimental assessment of the effects of process parameters on the microstructure and related properties of finished parts [11], rather than defining the optimal process parameters to obtain defect-free products. On the contrary, in some papers, finished parts are purposely fabricated under nonoptimal parameters to emphasize and detect the development of pores and other microstructural defects and reveal their effects on the performance of finished parts [12,13].

However, on account of the importance of reaching optimized processing conditions, especially when a new alloy grade must be handled, an increasing number of publications have addressed the rational analysis of manufacturing setup. In consequence, specific contributions have focused on process parameter optimization for some of the most common metal powders used in L-PBF. Some examples are listed in Table 1 [14,15,16,17,18,19,20,21,22,23,24,25]. However, each contribution is usually targeted to a specific feedstock and a specific PBF machine, so that at present a general optimization procedure is still missing. Moreover, the published papers are usually dedicated to bulk core geometries, and details are rarely provided about boundary, downskin and upskin processing, support fabrication, and special geometries, e.g., thin-walled constructs.

Noteworthy previous contributions have focused on the effect of the beam scanning strategy on the development of microstructural features. During L-PBF, energy is conveyed locally by the laser beam to the processed material, thus engendering steep temperature gradients and related thermal stresses or even plastic deformation. Cheng and colleagues [26] proposed a sequentially coupled 3D thermomechanical coupled finite element (FE) model as a tool for the choice of scanning strategy that minimizes part distortion. Uneven thermal distribution may also cause balling defects through Marangoni convection [27]. Numerical simulations originally applied to In718 have shown that the local temperature and consequent stress and deformation conditions in the built part are greatly dependent on the scanning pattern strategy [26]. Moreover, it has been proven that applying a parallel scanning strategy throughout the building part may result in overheating and consolidation problems. Spiraling patterns are expected to be beneficial for high-conductive materials, but they are unfeasible for nonconvex domains. An effective improvement may be achieved by scaling the conventional parallel scanning strategy down to small-area paintbrush or chessboard patterning in multiscan mode [27]. Recently, AlMangour et al. [28] pointed out that the laser scanning strategy directly influences the degree of densification and the mechanical anisotropy of fabricated parts. In fact, the scanning path controls the microstructural evolution through heat input and dissipation mechanisms. Generally speaking, rotating the scanning direction from one layer to the next interferes with the growth of defined columnar/epitaxial structures often encountered in L-PBF parts and blurs the individual laser tracks. The appropriate choice of scanning strategy may therefore result in preferential crystal growth or in more statistically distributed crystal orientations, corresponding to strongly anisotropic behavior or more uniform mechanical properties, respectively [28]. On the other hand, the process of double scanning and consequent remelting reduces the size of the melt pool and promotes the elimination of pores and defects. Since pores pin the movement of dislocations and boundaries that is responsible for recrystallization, the double scanning strategy promotes the process and causes the formation of finer dendrites [28].

The need to assess standard procedures is also demonstrated by the recent release of international standards dedicated to AM in general and PBF in more detail. Though not specifically addressed to process optimization, ISO/ASTM 52910:2018(E) describes point-by-point the requirements and recommendations that are useful to design parts and products to be produced by AM [29]. ASTM F3303-2018 specifies instead all the process characteristics and machine features that should be controlled in metal-based PBF in order to meet critical applications, especially in aerospace and medical components [30].

The drive to reach standard procedures is overwhelming whenever qualification of new materials and processing methods is involved [31,32]. To this aim, for example, Portolés et al. [33] contributed to establishing a qualification procedure to fabricate and repair aerospace parts produced by electron beam melting (EBM). The authors underlined the need for AM aerospace parts to comply with the existing regulations that cover both materials and fabrication methods, to answer the demanding requirements put forward by the aerospace industry, and to equal the high-quality performance offered by conventional technologies. As a first measure toward defining comprehensive quality assurance/quality control procedures, the methodology proposed by Portolés et al. [33] accounts for all the parameters that are expected to affect both the technical requirements for finished parts and the process reproducibility by means of nine steps or “studies” that span from the validation of recycled powder to the surface finish of built parts. The procedure defines the key variables to test within each study, fixes reference values for every single variable according to the requirements of international standards (wherever applicable), and proposes experimental procedures to perform the corresponding verification tests. However, it is worth noting that some of the nine studies described by Portolés et al. [33] are specific to aerospace part repair only. Moreover, the qualification procedure, as such, explains how to test feedstock materials and EBM parts in order to demonstrate their consistency and whether they conform to the directives for aerospace devices. As a consequence, the methodology is focused on how to substantiate the properties of materials and finished parts, rather than how to achieve optimal properties.

Even if the present contribution is intended as a proof of concept and not a definitive solution, it paves the way for the definition of a standard procedure to assess the most appropriate L-PBF processing conditions for new alloy grades, independent of the specific L-PBF appliance in use. As an additional advantage, though originally conceived for L-PBF, in principle the philosophy of the present procedure can be modified and adapted to EBM, too. The main intention is to foster a constructive discussion in the literature, in order to support the progressive buildup of an established and commonly accepted procedure to optimize PBF process parameters.

## 2. Method

### 2.1. General Outline

**Figure 1:**
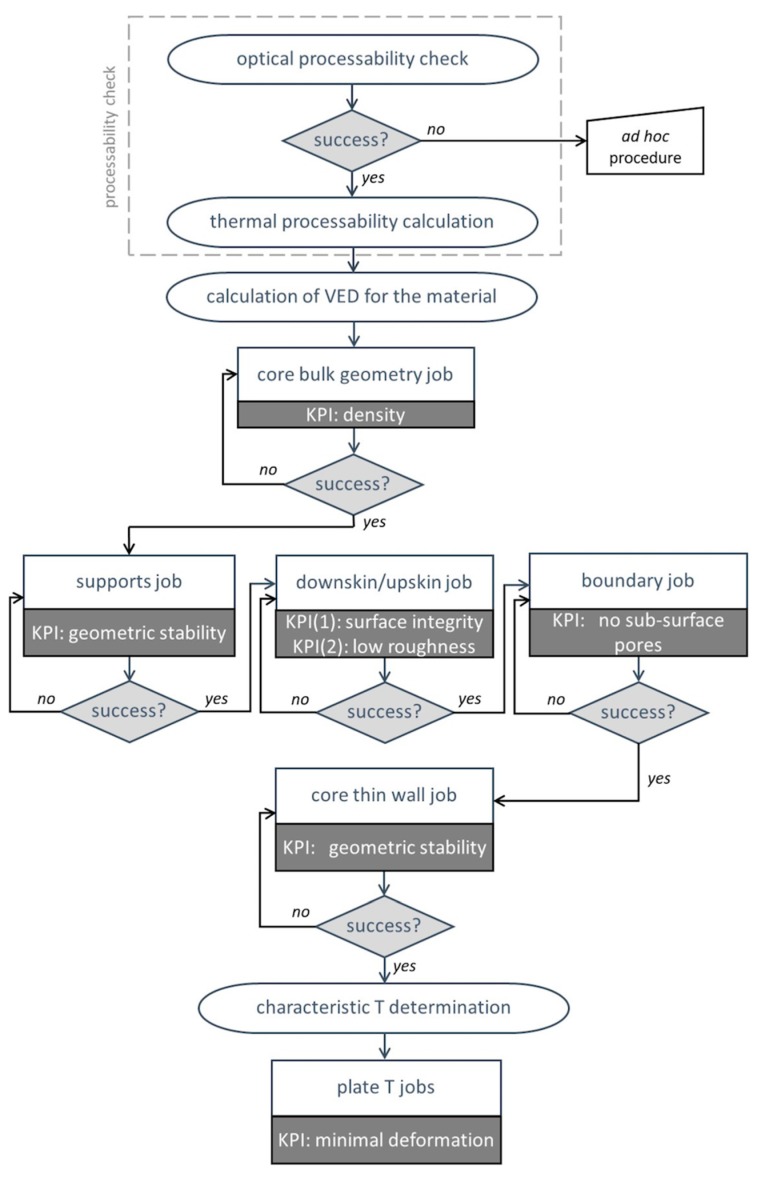
Flowchart showing the key points of the proposed optimization procedure. VED, volume energy density; KPI, key performance indicator.

The present method, which is developed to assess the proper parameters for processing a new alloy grade by L-PBF independent of the fabrication appliance in use and of the chemical composition of the feedstock powder, implies a multistep approach, including the following: general evaluation of the processability of the new material and investigation of core bulk geometry, supports, downskin/upskin, boundary, thin wall, and plate temperature. For each step, the method describes what parameters should be considered, what key performance indicators (KPIs) should be optimized, what geometry should be tested, and what measurement methods should be applied. Each point is the subject for a dedicated section in the following presentation, but a comprehensive outline is provided as a road map in Figure 1.

### 2.2. Processability Check

The processability of a material depends on two basic features: (i) the optical properties of the powder feedstock, especially its reflectivity to the laser beam, and (ii) the thermal behavior of the solidified material, which accounts for its thermal conductivity as well as its shrinkage during cooling.

As to the optical processability, the present method does not apply to high-reflectivity materials. In fact, for highly reflective materials, under unfortunate geometric positions, the reflected beam may be transmitted back through the beam delivery optics and into the laser source itself, potentially damaging the manufacturing equipment. Due to the uneven surface of the powder bed, the reflectivity *R_powder_* of the feedstock can be assumed to be around 70% of the reflectivity *R_bulk_* of the corresponding material in its bulk form [34]. If a standard laser source working at a wavelength of 1.06 µm is considered, a reflectivity threshold value of 0.95 for the bulk material (corresponding to around 0.67 for the powdered feedstock) can be fixed for the present procedure to be applicable. According to this definition, the present procedure is still pertinent to Al-based alloys that have a reflectivity *R_bulk_* close to 0.91 [35], but not to Cu-based feedstocks that have a reflectivity *R_bulk_* higher than 0.95 [36,37]. In principle, a material can be manufactured by means of L-PBF even if its reflectivity exceeds the proposed threshold value. However, in this case, the material must be investigated individually, by means of an *ad hoc* procedure, in order to obviate the risk of laser back-reflection damage.

Provided that *R_bulk_* does not exceed the aforementioned threshold value of 0.95, the thermal processability of the material under investigation must also be considered.

As a basic guideline, the thermal processability *TP* (W m^−1^·K^−1^) of the new material can be estimated as the ratio between its thermal conductivity, *k* (W m^−1^·K^−1^), and the linear shrinkage that the material experiences while cooling down from its melting temperature to room temperature. The shrinkage, in turn, can be roughly calculated by the product of the linear coefficient of thermal expansion of the material at the solid state, *α* (K^−1^), multiplied by the difference *ΔT* between the material’s melting temperature, *T_m_* (K), and room temperature, *T*_0_ (K). The change in specific volume that takes place during solidification can be disregarded, since it is not the cause of frozen stresses. *TP* can be expressed by Equation (1):

(1) $$TP=\frac{k}{\alpha \cdot \Delta T}$$

Some examples are provided in Table 2, where it is assumed *T*_0_ = 20 °C ≈ 293 K.

Even if the potential use of a new material in L-PBF depends on other relevant properties, including its weldability, and not just its thermal behavior, *TP* is intended here as a preliminary estimate for the ease with which the new material will be processed by L-PBF. In fact, if the thermal conductivity is low, the metal will remain locally in its molten state for a relatively long time, which is expected to impede the recoating operation of the subsequent powder layer. If the shrinkage is high, strong thermal deformations or even cracks are likely to occur, thus causing job failure [10]. As a consequence, for high values of *TP*, the material will be considered easily processable from the thermal point of view. Vice versa, if *TP* is low, the material should be classified as difficult to print. As a guideline, the procedure proposed here has been proven feasible for Inconel 625 but has not been verified yet for lower values of *TP*.

If the values for *k*, *T_m_*, and *α* are unknown for the new alloy being developed, as a first approximation the values for a similar grade can be used in place of the real ones.

### 2.3. Core Bulk Parameters

Determining the appropriate parameters to process core bulk geometries starts from evaluating the volume energy density, *VED* (J·mm^−3^), that is required to melt the processed material. A rough estimation of the *VED* that must be provided for consolidation through melting and subsequent solidification mechanisms can be based on the heat per unit volume, *q* (J·mm^−3^), that must be conveyed to the material to heat and melt it according to Equation (2):

(2) $$q=\left[c\cdot \Delta T+l_{f}\right]\rho$$

where *c* (J·kg^−1^·K^−1^), *l_f_* (J·kg^−1^), and *ρ* (kg·mm^−3^) are the specific heat capacity, specific latent heat of fusion, and density of the processed material, respectively. As already seen in Equation (1), Δ*T* is the difference between the melting temperature of the feedstock metal, *T_m_*, and room temperature, *T*_0_.

However, the *VED* must increase with respect to *q* through an efficiency coefficient *η* in order to account for various dissipative phenomena associated with L-PBF [40,41,42,43,44], including energy losses due to (i) the feedstock’s reflectivity, (ii) the cooling down of the incipient melt pool that occurs as a result of heat conduction through the powder bed, and (iii) additional losses. Accordingly, the overall efficiency *η* of the process is calculated here as the serial efficiency of three terms, and therefore the *VED* is obtained from *q* as shown in Equation (3). In the denominator, the first and second terms represent an attempt to estimate, from a physical standpoint, losses due to reflection and conduction, respectively. The third factor, η*, accounts for the remaining dissipative effects, especially the complex phenomena generated inside the melt pool. In fact, as described by Li et al. [43], the convective heat flux that is generated by turbulent flows inside the melt pool represents a significant term of the thermal balance, and again these flows are responsible for a reduction in L-PBF process efficiency. Moreover, it is important to highlight that the first two efficiency terms in the denominator of Equation (3) are based on the assumption of an ideal L-PBF process that neglects the interference that may occur, for example, between the laser beam and dust and splatters produced by the melt pool. It is acknowledged [45] that these interactions are responsible for two main effects on the laser behavior, power attenuation on the powder bed and slight defocusing of the laser beam. Unavoidably, this brings about additional losses. To conclude, the *VED* can be estimated from *q* according to Equation (3):

(3) $$VED=\frac{q}{\eta }=\frac{q}{\left(1-R_{powder}\right)\left(1-k_{rel}\right)\eta^{*}}$$

where *R_powder_* is the reflectivity of the powdered feedstock, expressed in fractional terms as before (0.70 × *R_met_*); *k_rel_* is the relative thermal conductivity of the powdered feedstock; and η* is the additional efficiency factor that, as a first approximation, can be assumed to be around 20% (η* ≈ 0.20).

In turn, *k_rel_* is calculated as the ratio between the thermal conductivity of the powdered feedstock *k_powder_* and that of pure silver in its bulk form, *k_Ag_*, as per Equation (4):

(4) $$k_{rel}=\frac{k_{powder}}{k_{Ag}}$$

The value of *k_powder_* value can be roughly estimated through a classical rule of mixtures [46] by assuming that the volume fraction of the metal powder *V_metal_* is 50% of the powder layer, as shown in Equation (5):

(5) $$k_{powder}=k_{metal}\cdot V_{metal}+k_{gas}\cdot (1-V_{metal})=0.5\cdot \left(k_{metal}+k_{gas}\right)\approx 0.5k_{metal}$$

where *k_metal_* and *k_gas_* are the thermal conductivity of the processed metal in its bulk form and that of the gas in the build chamber, respectively. It is worth noting that, due to the line-by-line scanning strategy adopted in PBF, one side of the scan track partly overlaps the previously solidified material, while the other side is still in contact with loose powder. As a consequence, strictly speaking, two different models should be applied to describe the thermal conductivity on the two sides of the melt track [47]. However, a full modelling of the thermal exchange mechanism is beyond the scope of the present contribution and, as a first approximation, only the thermal conductivity of the powder was considered. Though simplistic, this approach makes the calculations more straightforward and it goes for safety, since it describes the condition of the lowest thermal conductivity.

The required *VED*, as calculated by Equation (3), is provided by the laser beam through an appropriate setting of the process parameters as expressed in Equation (6) [48]:

(6) $$VED=\frac{P}{L\cdot h\cdot v}$$

where *P* (W) is the laser power, *L* (mm) is the layer thickness, *h* (mm) is the hatch distance, and *ν* (mm·s^−1^) is the laser scan speed for continuously operated lasers, to be substituted with the ratio between point-to-point distance, *P-T-P* (mm), and exposure time, *E_t_* (s), for lasers operated in pulsed mode.

AlMangour et al. [49], through a systematic change of laser scan speed, demonstrated that lower VED values reduce the temperature of the melt pool and increase the cooling rate. These conditions hinder grain growth, but also promote the development of large and interconnected pores that are associated with delamination and the formation of horizontal cracks. Remelting is also limited, with the consequence that texture intensity is reduced. On the other hand, high VED values produce continuous molten scan tracks and good metallurgical bonding between adjacent layers, but can also cause thermal stresses and the occurrence of spherical pores as a consequence of surface vaporization of the feedstock material. For very high VED values, a switch from conduction-mode welding to keyhole welding can be observed [49].

The value of the *VED* obtained from Equation (3) is taken as the medium point of a full factorial design of experiment (DOE), in which the *VED* must vary by ±30% in order to account for the approximations introduced so far. To that purpose, in Equation (6), *L* is considered as a constrained parameter imposed by the L-PBF appliance in use. The suggested variation range for *h* is 1.4 to 1.7 times the spot diameter of the laser beam. As a first attempt, the initial values for *P* can be centered on 90% of the maximum power of the machine. For lasers operated in pulsed mode, *P-T-P* can be set to equal the hatch distance. *v*, for continuously operated lasers, or *E_t_*, for pulsed lasers, must be determined accordingly. Since three variables (*h*, *P*, and *v* or *E_t_*) must be analyzed, a 3^3^ factorial design could be appropriate.

**Figure 2:**
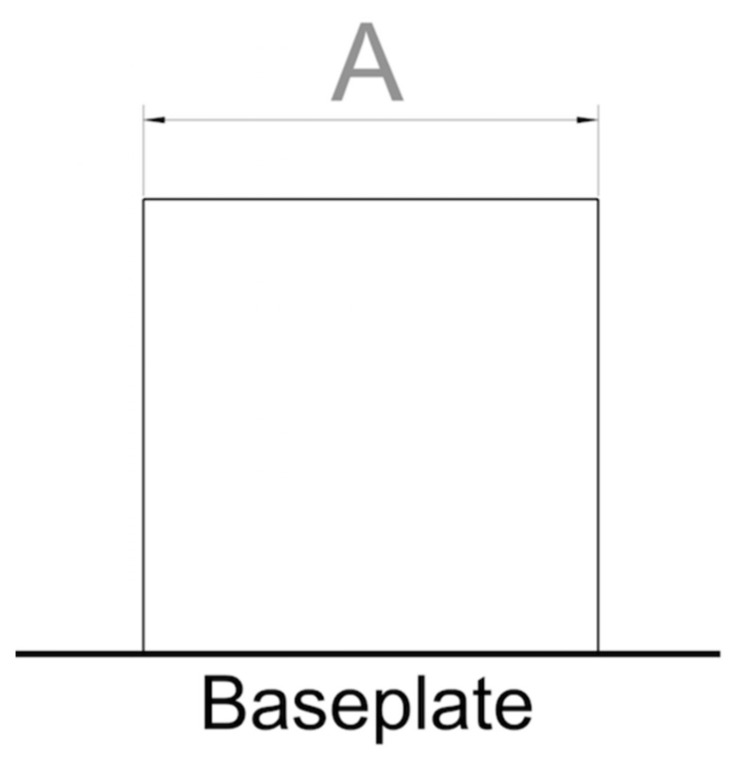
Recommended specimen geometry to be used for assessing core bulk parameters.

As displayed in Figure 2, the geometry proposed for assessing core bulk parameters is a cube with a side length of *A* (mm), where *A* is a suitable measure for embedding the specimens in resin and polishing them.

It is important to underline that the samples must be printed without any supports on their lower face, since there are no optimized parameters for supports yet. The cubes must instead be printed from the build plate directly. Since this is the very first printing attempt with the new material, some specimens may cause trouble during processing. As a consequence, the operator should supervise the build job as it progresses and remove those parts that hinder the recoater functionality.

The primary KPI for the core bulk geometry is density, and the secondary KPIs are melt pool shape and process productivity.

As to the primary KPI, the job is considered successful if the density of at least one specimen reaches 99.5%. Various strategies have been discussed in the literature to determine the density of L-PBF parts. Basically, density can be evaluated by x-ray computed microtomography, by Archimedes’ method, and by image analysis. X-ray microtomography is a valuable nondestructive method that gives information about size, shape, and distribution of pores. As a drawback, specific equipment is required to perform the test. Additionally, the data acquisition may be time-consuming and the interpretation not straightforward, because a standard method is still missing for this purpose [50,51]. In order to obtain a quick measure of the amount of porosity present in finished parts, as required in this case, Archimedes’ method and image analysis may suffice. Nonetheless, the former approach cannot reveal the presence of defects associated with the lack of fusion, since nonconsolidated particles affect the mechanical behavior but not the density of finished parts [12]. As a consequence, image analysis may be considered the measurement technique of choice for the present procedure to evaluate the amount of porosity and hence the density of the finished part under investigation.

On the other hand, the metallographic preparation of cross-sections parallel to the growth direction is also necessary to investigate the melt pool. The printing parameters’ assessment can be considered satisfactory if the melt pool is consistent throughout the cross-section, no keyhole is observed, and the width of the melt pool matches the hatch distance and the depth involves two or, at most, three layers. If more than one sample achieves satisfactory results in terms of density and melt pool characteristics, productivity should be privileged to choose the right set of parameters.

If none of the built parts meets the proposed requirements for the KPIs, the job must be repeated by designing a new DOE shifted toward the most performing parameters of the first run.

### 2.4. Support Parameters

The present procedure preferentially applies to block supports, because they are the most widely diffused supports in L-PBF [17].

In order to define the support parameters, the first step requires determining whether supports must be scanned layer-by-layer or every second layer. If feasible, the latter solution would be preferable to optimize productivity and facilitate the removal operations. Nonetheless, the former printing strategy becomes compulsory for materials with poor processability (i.e., materials that are classified as difficult to print according to the *TP* check) to compensate for potential issues related to bad heat exchange or residual stresses.

In general terms, the approach to determining support parameters is similar to that already developed for core bulk geometry. However, it is worth noting that block supports are commonly scanned with only one laser pass so that their wall thickness corresponds to the melt pool width. Since there is no hatching when block supports are involved, the energy input is preferentially expressed through the surface energy density, *SED*, instead of the volume energy density, *VED*, as previously seen for bulk geometries. For continuous lasers, the *SED* is calculated as shown in Equation (7):

(7) $$SED=\frac{P}{n\cdot L\cdot v}$$

where *n* = 1 if supports are scanned layer-by-layer and *n* = 2 if supports are scanned every second layer. As already discussed in Section 2.3, *L* can be considered as a fixed parameter and *P* can initially vary around 90% of the maximum power allowed by the L-PBF machine in use. *v* is then calculated accordingly. For pulsed lasers, *v* should be replaced by the ratio between *P-T-P* and *E_t_*.

From a practical standpoint, a DOE must be planned in which the medium value of the *SED* equals the value of the *VED* calculated by Equation (3) multiplied by the laser spot diameter. The suggested variation range for the *SED* is ±20% to account for the approximations that have been introduced. Since two variables (*P* and *v* or *E_t_*) must be analyzed, a 3^2^ factorial design could be appropriate.

**Figure 3:**
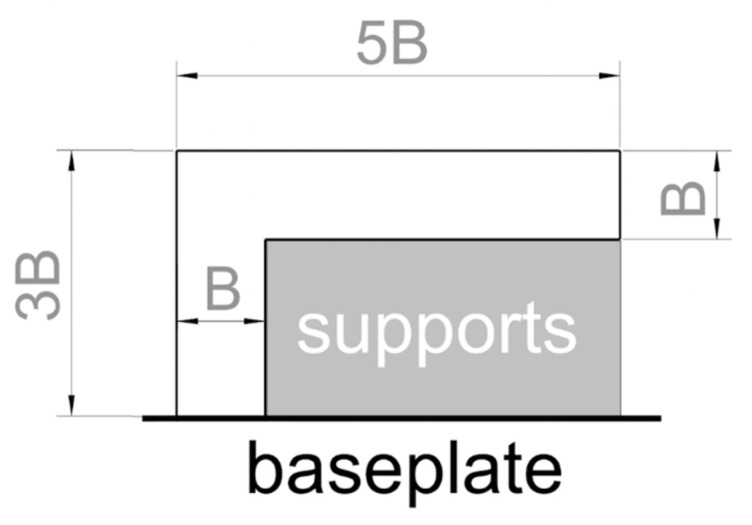
Recommended specimen geometry to be used for the assessment of support parameters.

The recommended L-shaped geometry for assessing the support parameters is shown in Figure 3, where *B* is approximately twice as much as the *XY* grid dimension of the block supports, where *XY* is the plane of powder deposition. *B* is also the thickness of the sample. If the geometry in Figure 3 is actually adopted to assess the support parameters, the supports must be generated on the indicated surface.

To optimize the support parameters, the primary KPI is geometric stability, meaning that the obtained bulk supports must not rip apart from the base plate or warp. In more detail, given the really demanding geometry of the reference specimen in Figure 3 that is purposely designed to emphasize the effect of supports, moderate warpage is acceptable provided that it does not interfere with the recoater. The secondary KPI for support parameters is productivity.

As a consequence, the job is successfully concluded if at least one specimen is geometrically stable. If multiple specimens satisfy the geometric requirements, the one with the highest productivity must be preferred.

### 2.5. Downskin and Upskin Parameters

Only one job is needed to determine both the downskin and upskin parameters at the same time. In principle, the approach detailed in Section 2.3 for core bulk geometries can be extended to the definition of downskin and upskin parameters, too. Nevertheless, this time the *VED* values to be used as the medium points for the DOEs must be 50% and 80% of the *VED* value calculated according to Equation (3) if the downskin and upskin parameters, respectively, are considered. The suggested variation range for the *VED* is ±20% in both cases. Moreover, for the upskin parameters, a reduction of 20% in hatch distance is suggested with respect to the previously determined core bulk parameters. As already seen for the *VED* in Section 2.3, a 3^3^ factorial design could be used to assess the three variables involved (*h*, *P*, and *v* or *E_t_*).

**Figure 4:**
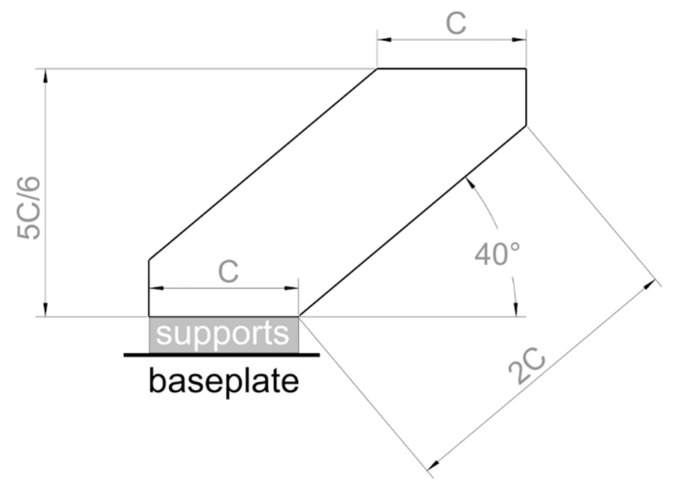
Recommended specimen geometry to be used for assessing downskin and upskin parameters.

The reference geometry for this job is shown in Figure 4. The proposed model has a twofold purpose, as it was designed to (i) allow for use of the downskin and upskin parameters on horizontal faces, and (ii) simultaneously verify the applicability of the core bulk parameters established before to down-facing and up-facing angled surfaces. In fact, since the boundary parameters have not been analyzed yet, it is important to underline that, apart from the two horizontal faces, the whole specimen should be built using core parameters only. It is worth noting that the present procedure does not account for the possibility of applying the downskin and upskin parameters to oblique faces. In the reference geometry of Figure 4, the value for *C* should be at least four times as much as the grid dimension used for the block supports. At a minimum, *C* must be chosen in such a way that the downskin surface is large enough for the statistical investigation of cracks to be carried out. *C* can be assumed as the depth of the sample, too.

The primary KPI for the downskin parameters is the integrity of the lower surface of the specimen as observed under the optical microscope on metallographic sections, including the absence of (i) semidetached particles, (ii) cracks, and (iii) subsurface pores. In order to verify these conditions, the samples must be cut and polished on a plane parallel to the face shown in Figure 4. The aforementioned defects should be monitored carefully, since they can induce failure mechanisms. Besides, the minimal deformation of the layer being processed with the downskin parameters should be selected as the secondary KPI and closely controlled, since it affects the feasibility of complex geometries. This characteristic can be verified in real time through the layer control system of the L-PBF equipment.

The primary KPI for the upskin parameters is surface roughness, which must be minimized. Moreover, as the secondary KPI, the depth of the melt pool on the upper surface must not exceed the thickness of two consolidated layers, as already prescribed in Section 2.3 for core bulk geometries.

### 2.6. Boundary Parameters

As mentioned in Section 2.4 for the assessment of support parameters, the *SED* should be considered instead of the *VED* every time a geometry is scanned with a single pass. This applies to the boundary, too. The *SED* value to be used as the medium point for the DOE should be calculated as detailed in Equation (7) by assuming *n* = 1 and introducing the values for *P* and *v* previously optimized for core bulk geometries (Section 2.3). The reference value obtained in this way must vary by ±80% in a two-factorial design, for example on three levels, in order to optimize *P* and *v*.

**Figure 5:**
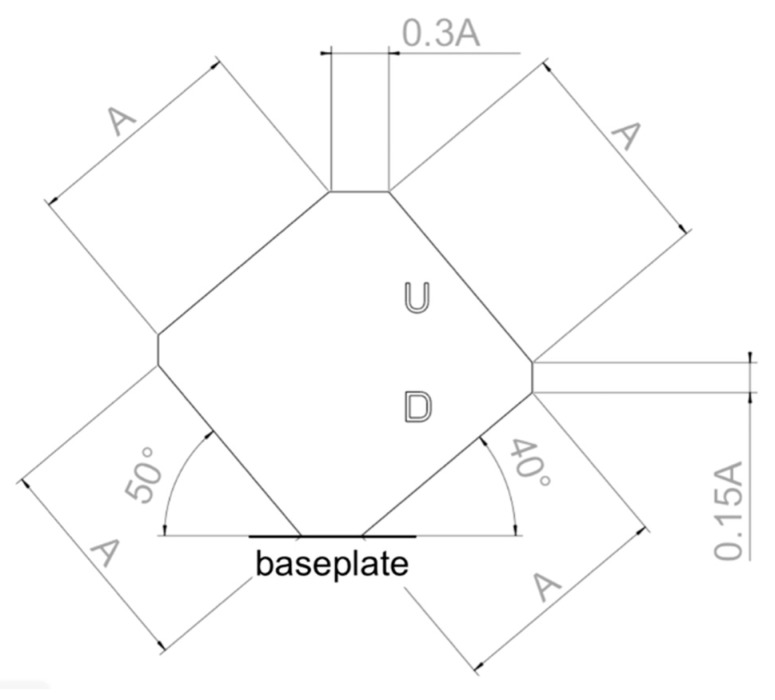
Recommended specimen geometry to be used for assessing boundary parameters.

The geometry to be used in this job, as shown in Figure 5, is meant to emphasize the effect of the boundary parameters in down-facing angled surfaces, vertical surfaces, and up-facing angled surfaces, which are labelled in Figure 5 as *DF*, *V*, and *UF*, respectively. It is worth noting that, for the typical values of self-supporting angles in metal L-PBF, the *DF* surface in Figure 5 has been designed to represent an extreme condition. In this geometry, the dimension for *D* should be fixed in such a way that the roughness can be easily measured. The same *D* value can be applied to the depth of the specimen, too.

The primary KPI for the boundary parameters is the absence of subsurface porosity, that is to say, the absence of pores where the transition between core and boundary occurs. In order to verify this condition, the samples must be cut and polished on a plane parallel to the face shown in Figure 5 and the subsurface zone should be investigated close to the *DF*, *V*, and *UF* sides.

Roughness should be considered as the secondary KPI for this job. Subsequently, if two or more sets of parameters achieve equally good KPIs, priority should be given to the highest productivity.

### 2.7. Thin Wall Parameters

This step of the procedure is done to verify the suitability of the parameters previously identified for the core bulk geometry and the boundary when a thin wall part has to be printed.

A DOE should be developed where the medium point is set on the optimum parameters identified for the core geometry in Section 2.3. and the boundary in Section 2.6. The suggested variation for the *VED* and *SED* values is ±15%.

**Figure 6:**
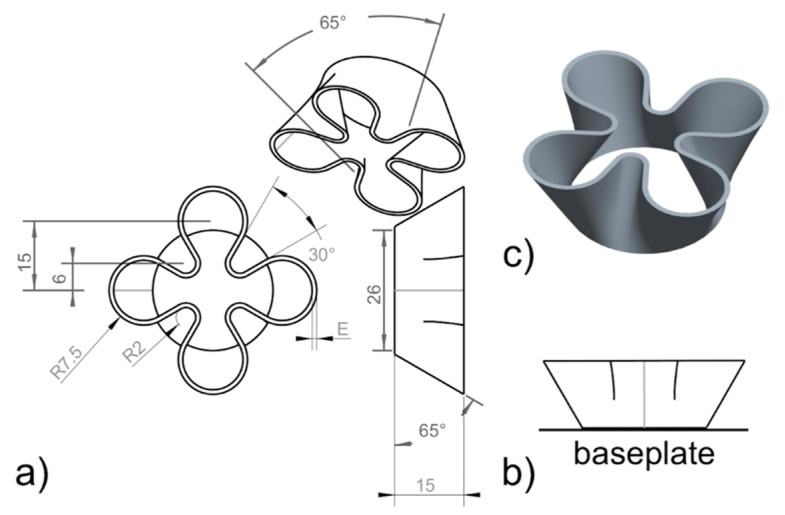
Recommended specimen geometry to be used for assessing thin-wall geometry: (**a**) model and dimensions; (**b**) positioning on baseplate; (**c**) 3D rendering.

The reference geometry in Figure 6 has been specifically designed for the purpose. *E* is the wall thickness of the specimen, and its value depends on the thermal processability of the material. As a general guideline, a wall thickness *E* of 0.8 mm can be assumed for materials with *TP* at least in the range of 600 (W·m^−^^1^·K^−1^). Otherwise, *E* should not be less than 1.2 mm.

The KPI for the thin-wall job is the geometric stability of the sample. The geometry in Figure 6 was conceived to highlight several possible processing issues, including shrinkage-related deformation of both down- and up-facing angled thin walls and deformation caused by the mechanical interplay with the recoater. In this regard, the job should be monitored continuously to prevent damage to the recoater.

It is also important that the transition between core bulk and boundary zones is as smooth as previously observed in boundary specimens.

### 2.8. Plate Temperature

In principle, more than one job is needed to find an optimal base plate temperature. To that aim, it is recommended to process at least two jobs at different plate temperatures.

As shown in Equation (8), two factors decide on the highest temperature to test, *T_high_* (K): (i) the upper limit that the machine can reach, *T_machine_* (K), and (ii) the aging temperature (or relieving temperature) of the alloy that is being printed, *T_ageing_* (K):

(8) $$T_{high}=\min\{T_{machine};\left(T_{ageing}-20\right)\}$$

Given the number of jobs (at least two) that can be dedicated to this study, uniformly distributed values of the plate temperature should be considered in the range (*T*_0_, *T_high_*).

**Figure 7:**
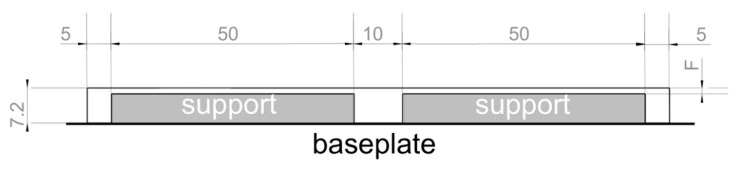
Recommended specimen geometry to be used for assessing plate temperature.

The same geometry shown in Figure 7 must be built in all jobs. Support structures must be placed only where specified in Figure 7, whereas the middle thick volume and two peripheral ones must be directly welded to the baseplate. The distance *F* in Figure 7 is an arbitrary value ranging from 1.2 mm to 4 mm. However, it is worth mentioning that stronger deformations are expected for greater *F* values. As a consequence, relatively low values of *F* should be preferred for those alloys that exhibit high shrinkage, and vice versa.

After each job, the samples must be partly cut away from the build plate by means of wire electrodischarge machining (EDM). In more detail, the central portion of the part must remain attached to the build plate, which implies that two separate cuts are needed on the sides. As an important recommendation, identical samples must be built in different areas of the build plate, so that any difference caused by local temperature inequalities can be noted.

The KPI to choose the best plate temperature is *Z* deformation after cutting: a higher deformation indicates stronger residual stresses in the part.

## 3. Application Hints and Final Remarks

In order to consolidate the proposed method and to sum up how it applies to a specific test case, the alloy Inconel 625 is taken as a reference. Table 3 lists all the material properties of Inconel 625 that are needed as a starting point for the procedure and reports the calculation of the medium point and related variation ranges for the *VED* and *SED* values at each step of the procedure as outlined in Figure 1 and described in Section 2.3, Section 2.4, Section 2.5, Section 2.6, Section 2.7 and Section 2.8. The step-by-step investigation of the development of Inconel 625 processing parameters based on the present method will be the subject of a dedicated contribution.

As a final remark, the paper proposes an exploratory standardization of the procedure to follow to develop a complete set of process parameters for L-PBF of innovative alloys, new machines, or untested alloy/machine combinations. Even though the outlined strategy is still preliminary, it takes into account all the laser/material interactions in different local geometries of the build and comprises, for each possible interaction, a specific geometry for test specimens, standard energy parameters to be analyzed through a design of experiment, and measurable key performance indicators. The method aims to foster scientific debate toward the accomplishment of shared standards.

## Figures and Tables

**Table 1:** Representative literature on process parameter optimization for specific powders.

Metal of Interest	Target of Optimization	Reference
Waspaloy	3D parts	[14]
Nickel	3D parts	[15]
Maraging steel	3D parts	[16]
17-4 PH	Support structures	[17]
304 L and 904 L	Single tracks	[18]
CoCrMo	Single tracks	[19]
Ti-6Al-4V	Single tracks	[20]
Ti-6Al-4V	3D parts	[21]
Ti-5Al-2.5Sn	From single tracks to 3D parts	[22]
AlSi10Mg	Single tracks	[23]
AlSi10Mg	From single tracks to single layers	[24]
Al-Cu-Mg alloy	From single tracks to 3D parts	[25]

**Table 2:** Characteristic values for *k*, *α*, and *ΔT = T_m_ − T*_0_ and corresponding evaluation of *TP* for some metals of interest in laser-based powder bed fusion (L-PBF) [38,39].

Material	*k*	*α*	Δ*T* = *T_m_* − *T*_0_	*TP* = *k*/(*α* ×Δ*T*)
	(W·m^−1^·K^−1^)	(K^−^^1^)	(K)	(W m^−1^ K^−1^)
AlSi10 [38]	113	22 × 10^−6^	570	≈9000
Ti-6Al-4V [38]	6.7	9.2 × 10^−6^	1635	≈445
Inconel 625 [39]	9.8	15 × 10^−6^	1330	≈490
AISI316 [39]	16.2	16.2 × 10^−6^	1380	≈725
Hastelloy X [39]	9.1	16 × 10^−6^	1335	≈430
Cu [38]	391	17.7 × 10^−6^	1060	≈21,000

**Table 3:** Material properties of Inconel 625 [39,52,53], processability check, and calculation of the VED and surface energy density (SED) values and related variation ranges. DOE, design of experiment; t.b.d., to be determined.

Material Properties						
*R_bulk_*	0.70 (70%)					
k (W·m^−1^·K^−1^)	9.8					
*α* (K^−1^)	15 × 10^−6^					
*T_m_* (K)	1623					
*c* (J·kg^−1^·K^−1^)	410					
*l_f_* (J·kg^−1^)	227 × 10^3^					
*ρ* (kg·mm^−3^)	8.44 × 10^−6^					
**Technical Specifications**						
Laser spot diameter (μm)	55					
**Processability check**						
Optical processability check	*R_bulk_* < 0.95		*verified*			
Thermal processability check	*TP* = 490 (W·m^−1^·K^−1^)		*verified*			
**Calculation of the *VED***						
Calculation of *q* (J·mm^−3^)	6.58					
Estimate of *ƞ*	0.10 (10%)					
*VED* (J·mm^−3^)	64					
**Calculation of the *SED***						
*SED* (J·mm^−2^)	3.5					
**Parameter Optimization**						
	*VED_min_*	*VED_medium_*	*VED_max_*	*SED_min_*	*SED_medium_*	*SED_max_*
	(J·mm^−3^)	(J·mm^−3^)	(J·mm^−3^)	(J·mm^−2^)	(J·mm^−2^)	(J·mm^−2^)
CORE BULK DOE	49	64	83			
SUPPORTS DOE				2.9	3.5	4.2
DOWNSKIN DOE	27	32	38			
UPSKIN DOE	42	51	61			
BOUNDARY DOE				*SED_medium_* −80%	t.b.d. based on CORE BULK DOE	*SED_medium_* +80%
THIN WALL DOE						
Core parameters	*VED_medium_* −15%	t.b.d. based on CORE BULK DOE	*VED_medium_* +15%			
Boundary parameters				*SED_medium_* −15%	t.b.d. based on BOUNDARY DOE	*SED_medium_* +15%
PLATE TEMPERATURE Jobs						
	Calculation of *T_high_*					
	Calculation of temperatures to be tested					

## References

[1] Petrovic V., Vicente Haro Gonzalez J., Jorda Ferrando O., Delgado Gordillo J., Ramon Blasco Puchades J., Portolès Griñan L. (2011). Additive layered manufacturing: Sectors of industrial application shown through case studies. Int. J. Prod. Res..

[2] Linxi Z., Quanzhan Y., Guirong Z., Fangxin Z., Gang S., Bo Y. (2014). Additive manufacturing technologies of porous metal implants. China Foundry.

[3] Gibson I., Rosen D.W., Stucker B. (2010). Additive Manufacturing Technologies. Rapid Prototyping to Direct Digital Manufacturing.

[4] Atzeni E., Iuliano L., Marchiandi G., Minetola P., Salmi A., Bassoli E., Denti L., Gatto A. Additive manufacturing as a cost-effective way to produce metal parts. High Value Manufacturing: Advanced Research in Virtual and Rapid Prototyping. Proceedings of the 6th International Conference on Advanced Research in Virtual and Rapid Prototyping.

[5] Bassoli E., Sewell N., Denti L., Gatto A. (2013). Investigation into the failure of Inconel exhaust collector produced by laser consolidation. Eng. Fail. Anal..

[6] Girardin E., Barucca G., Mengucci P., Fiori F., Bassoli E., Gatto A., Iuliano L., Rutkowski B. (2016). Biomedical Co-Cr-Mo components produced by Direct Metal Laser Sintering. Mater. Today Proc..

[7] Grasso M., Colosimo B.M. (2017). Process defects and in situ monitoring methods in metal powder bed fusion: A review. Meas. Sci. Technol..

[8] Mani M., Lane B., Donmez A., Feng S., Moylan S., Fesperman R. Measurement Science Needs for Real-Time Control of Additive Manufacturing Powder Bed Fusion Processes, NISTIR 8036.

[9] Baturynska I., Semeniuta O., Martinsen K. (2018). Optimization of process parameters for powder bed fusion additive manufacturing by combination of machine learning and finite element method: A conceptual framework. Procedia CIRP.

[10] Vlasea M.L., Lane B.M., Lopez F.F., Mekhontsev S., Donmez M.A. Development of powder bed fusion additive manufacturing test bed for enhanced real time process control. Proceedings of the International Solid Freeform Fabrication Symposium.

[11] Thijs L., Verhaeghe F., Craeghs T., Van Humbeeck J., Kruth J.-P. (2010). A study of the microstructural evolution during selective laser melting of Ti-6Al-4V. Acta Mater..

[12] Gong H., Rafi K., Gu H., Janaki Ram G.D., Starr T., Stucker B. (2015). Influence of defects on mechanical properties of Ti–6Al–4 V components produced by selective laser melting and electron beam melting. Mater. Des..

[13] Tang M., Pistorius P.C. (2017). Oxides, porosity and fatigue performance of AlSi_10_Mg parts produced by selective laser melting. Int. J. Fatigue.

[14] Mumtaz K.A., Erasenthiran P., Hopkinson N. (2008). High density selective laser melting of Waspaloy^®^. J. Mater. Process. Technol..

[15] Yap C.Y., Tan H.K., Du Z., Chua C.K., Dong Z. (2017). Selective laser melting of nickel powder. Rapid Prototyp. J..

[16] Casalino G., Campanelli S.L., Contuzzi N., Ludovico A.D. (2015). Experimental investigation and statistical optimisation of the selective laser melting process of a maraging steel. Opt. Laser Technol..

[17] Morgan D., Agba E., Hill C. (2017). Support structure development and initial results for metal powder bed fusion additive manufacturing. Procedia Manuf..

[18] Yadroitsev I., Gusarov A., Yadroitsava I., Smurov I. (2010). Single track formation in selective laser melting of metal powders. J. Mater. Process. Technol..

[19] Ciurana J., Hernandez L., Delgado J. (2013). Energy density analysis on single tracks formed by selective laser melting with CoCrMo powder material. Int. J. Adv. Manuf. Technol..

[20] Song B., Dong S., Zhang B., Liao H., Coddet C. (2012). Effects of processing parameters on microstructure and mechanical property of selective laser melted Ti6Al4V. Mater. Des..

[21] Wang Y.M., Kamath C., Voisin T., Li Z. (2017). A processing diagram for high-density Ti-6Al-4V by selective laser melting. Rapid Prototyping J..

[22] Wei K., Wang Z., Zeng X. (2017). Preliminary investigation on selective laser melting of Ti-5Al-2.5Sn-Ti alloy: From single tracks to bulk 3D components. J. Mater. Process. Technol..

[23] Kempen K., Thijs L., Van Humbeeck J., Kruth J.P. (2014). Processing AlSi_10_Mg by selective laser melting: Parameter optimisation and material characterisation. Mater. Sci. Technol..

[24] Aboulkhair N.T., Maskery I., Tuck C., Ashcroft I., Everitt N.M. (2016). On the formation of AlSi_10_Mg single tracks and layers in selective laser melting: Microstructure and nano-mechanical properties. J. Mater. Process. Technol..

[25] Nie X., Zhang H., Zhu H., Hu Z., Ke L., Zeng X. (2018). Analysis of processing parameters and characteristics of selective laser melted high strength Al-Cu-Mg alloys: From single tracks to cubic samples. J. Mater. Process. Technol..

[26] Cheng B., Shrestha S., Chou K. (2016). Stress and deformation evaluations of scanning strategy effect in selective laser melting. Addit. Manuf..

[27] Jhabvala J., Boillat E., Antignac T., Glardon R. (2010). On the effect of scanning strategies in the selective laser melting process. Virtual Phys. Prototyp..

[28] AlMangour B., Grzesiak D., Yang J.-M. (2017). Scanning strategies for texture and anisotropy tailoring during selective laser melting of TiC/316L stainless steel nanocomposites. J. Alloy Compd..

[29] ISO/ASTM 52910:2018(E) (2018). Additive Manufacturing—Design—Requirements, Guidelines and Recommendations.

[30] ASTM F3303-18 (2018). Standard for Additive Manufacturing—Process Characteristics and Performance: Practice for Metal Powder Bed Fusion Process to Meet Critical Applications.

[31] Frazier W.E., Polakovics D., Koegel W. (2001). Qualifying of metallic materials and structures for aerospace applications. JOM J. Miner. Metals Mater. Soc..

[32] Brice C.A., Allison J., Collins P., Spanos G. (2011). Unintended consequences: How qualification constraints innovation. 1st World Congress on Integrated Computational Materials Engineering, TMS (The Minerals, Metals & Materials Society), 2011.

[33] Portolés L., Jordá O., Jordá L., Uriondo A., Esperon-Migueza M., Perinpanayagam S. (2016). A qualification procedure to manufacture and repair aerospace parts with electron beam melting. J. Manuf. Syst..

[34] Boley C.D., Khairallah S.A., Rubenchik A.M. (2015). Calculation of laser absorption by metal powders in additive manufacturing. Appl. Optics.

[35] Aboulkhair N.T., Everitt N.M., Ashcroft I., Tuck C. (2014). Reducing porosity in AlSi_10_Mg parts processed by selective laser melting. Addit. Manuf..

[36] Naeem M. (2013). Laser processing of reflective materials. Laser Tech. J..

[37] Zavala-Arredondo M., Boone N., Willmott J., Childs D.T.D., Ivanov P., Groom K.M., Mumtaz K. (2017). Laser diode area melting for high speed additive manufacturing of metallic components. Mater. Des..

[38] (1990). ASM Handbook Volume 2: Properties and Selection: Nonferrous Alloys and Special-Purpose Materials.

[39] (1990). ASM Handbook Volume 1: Properties and Selection: Irons, Steels, and High-Performance Alloys.

[40] Carter L.N., Wang X., Read N., Khan R., Aristizabal M., Essa K., Attallah M.M. (2016). Process optimisation of selective laser melting using energy density model for nickel based superalloys. Mater. Sci. Technol..

[41] Cheng B., Chou K. Melt pool evolution study in selective laser melting. Proceedings of the International Solid Freeform Fabrication Symposium.

[42] Gürtler F.-J., Karg M., Leitz K.-H., Schmidt M. (2013). Simulation of laser beam melting of steel powders using the three-dimensional volume of fluid method. Phys. Proc..

[43] Li L., Lough C., Replogle A., Bristow D., Landers R., Kinzel E. Thermal modeling of 304L stainless steel selective laser melting. Proceedings of the ASME 2017 International Mechanical Engineering Congress and Exposition, Advanced Manufacturing.

[44] Thompson S.M., Bian L., Shamsaei N., Yadollahi A. (2015). An overview of Direct Laser Deposition for additive manufacturing; Part I: Transport phenomena, modeling and diagnostics. Addit. Manuf..

[45] Galba M.J., Reischle M., Bandyopadhyay A., Bose S. (2016). Additive manufacturing of metals using powder-based technology. Chapter 4: Additive Manufacturing.

[46] Jopek H., Strek T., Ahsan A. (2011). Optimization of the effective thermal conductivity of a composite. Convection and Conduction Heat Transfer.

[47] Bauereiß A., Scharowsky T., Körner C. (2014). Defect generation and propagation mechanism during additive manufacturing by selective beam melting. J. Mater. Process. Technol..

[48] Yang K.V., Rometsch P., Jarvis T., Rao J., Cao S., Davies C., Wu X. (2018). Porosity formation mechanisms and fatigue response in Al-Si-Mg alloys made by selective laser melting. Mater. Sci. Eng. A.

[49] AlMangour B., Grzesiak D., Borkar T., Yang J.-M. (2018). Densification behavior, microstructural evolution, and mechanical properties of TiC/316L stainless steel nanocomposites fabricated by selective laser melting. Mater. Des..

[50] Kim F.H., Moylan S.P., Garboczi E.J., Slotwinski J.A. (2017). Investigation of pore structure in cobalt chrome additively manufactured parts using X-ray computed tomography and three-dimensional image analysis. Addit. Manuf..

[51] Maskery I., Aboulkhair N.T., Corfield M.R., Tuck C., Clare A.T., Leach R.K., Wildman R.D., Ashcroft I.A., Hague R.J.M. (2016). Quantification and characterisation of porosity in selectively laser melted Al–Si_10_–Mg using X-ray computed tomography. Mater. Charact..

[52] Criales L.E., Arısoy Y.M., Özel T. (2016). Sensitivity analysis of material and process parameters in finite element modeling of selective laser melting of Inconel 625. Int. J. Adv. Manuf. Technol..

[53] Liu L., Hirose A., Kobayashi K.F. (2002). A numerical approach for predicting laser surface annealing process of Inconel 718. Acta Mater..

